# Supplementary material for: The effect of diaphragmatic breathing and diaphragmatic mobilization on physical performance, fear of falling, and quality of life in community-dwelling older adults: A randomized controlled trial
Source: PLoS One. 2026 Jan 5;21(1):e0339868. doi: 10.1371/journal.pone.0339868 (PMC12768353; doi:10.1371/journal.pone.0339868)
Supplement: S1 Table — This table summarizes the demographic characteristics of all participants across the three groups. No statistically significant differences were found in any demographic variable among the groups (p > 0.05), indicating that participants had similar prognostic factors at baseline. (DOCX) [file pone.0339868.s001.docx]

**S1 Table**

**Characteristics for enrolled participants who started treatment (N=54).**

|  |  | **Treatment Group** | | |  |
| --- | --- | --- | --- | --- | --- |
| **Characteristic** | **Complete sample (N=54)** | **DB (n=18)** | **DB+DM (n=18)** | **Control (n=18)** | **P value** |
| **Age (year)** | 68.33 ± 3.40 | 68.72 ± 3.59 | 68.00 ± 3.51 | 68.28 ± 3.27 | 0.82 |
| **Weight (kg)** | 61.92 ± 6.54 | 62.88 ± 7.05 | 60.61 ± 5.83 | 62.28 ± 6.84 | 0.57 |
| **Height (m)** | 1.60 ± 0.08 | 1.62 ± 0.09 | 1.59 ± 0.07 | 1.60 ± 0.08 | 0.72 |
| **BMI (kg/m^2^)** | 23.98 ± 1.00 | 24.02 ± 1.01 | 23.81 ± 1.09 | 24.10 ± 0.93 | 0.67 |
| **Sex** | | | | | |
| *Female, n (%)* | 28 (52) | 8 (44) | 10 (56) | 8 (44) | 0.75 |
| *Male, n (%)* | 26 (48) | 10 (56) | 8 (44) | 10 (56) |  |

Note: Data are presented as mean ± SD

Abbreviations: DB = Diaphragmatic Breathing; DM = Diaphragmatic Mobilization
