# Supplementary material for: The effect of diaphragmatic breathing and diaphragmatic mobilization on physical performance, fear of falling, and quality of life in community-dwelling older adults: A randomized controlled trial
Source: PLoS One. 2026 Jan 5;21(1):e0339868. doi: 10.1371/journal.pone.0339868 (PMC12768353; doi:10.1371/journal.pone.0339868)
Supplement: S2 Table — This table presents the results of the group-by-time interaction for all primary and secondary outcome measures. A significant interaction effect was observed for all outcomes except for the Mental Health (MH) and Social Functioning (SF) domains of SF-36. (DOCX) [file pone.0339868.s002.docx]

**S2 Table**

**Overall statistical results of all outcome measures based on mixed ANOVA.**

| **Outcome measures** | **Main effect (Group)** | | | **Main effect (time)** | | | **Interaction (group * time)** | | |
| --- | --- | --- | --- | --- | --- | --- | --- | --- | --- |
|  | **F (df)** | **η²** | **P- value** | **F (df)** | ***η²*** | **P- value** | **F (df)** | **η²** | **P- value** |
| **mini-BEST (points)** | 5.00 (2, 51) | 0.164 | **0.010** | 1.06 (2, 102) | 0.020 | 0.341 | 16.23 (4, 102) | 0.389 | **<0.001** |
| **TUG (sec)** | 2.69 (2, 51) | 0.095 | 0.077 | 12.18 (2, 102) | 0.193 | **<0.001** | 9.86 (4, 102) | 0.279 | **<0.001** |
| **GV (m/sec)** | 35.18 (2, 51) | 0.580 | **<0.001** | 91.27 (2, 102) | 0.642 | **<0.001** | 41.56 (4, 102) | 0.619 | **<0.001** |
| **5xSTS (sec)** | 0.57 (2, 51) | 0.022 | 0.567 | 1.88 (2, 102) | 0.036 | 0.165 | 4.97 (4, 102) | 0.163 | **0.001** |
| **ABC (%)** | 25.59 (2, 51) | 0.501 | **<0.001** | 100.04 (2, 102) | 0.662 | **<0.001** | 61.33 (4, 102) | 0.706 | **<0.001** |
| **FSS (score)** | 44.53 (2, 51) | 0.636 | **<0.001** | 78.78 (2, 102) | 0.607 | **<0.001** | 35.85 (4, 102) | 0.584 | **<0.001** |
| **SF-36 (score)** | | | | | | | | | |
| *Physical function* | 3.206 (2, 51) | 0.112 | **0.049** | 5.978 (2, 102) | 0.105 | **0.018** | 6.819 (4, 102) | 0.211 | **0.002** |
| *Role limits – Physical* | 3.597 (2, 51) | 0.124 | **0.035** | 9.928 (2, 102) | 0.163 | **< 0.001** | 2.584 (4, 102) | 0.092 | **0.041** |
| *Role limits – Emotional* | 1.545 (2, 51) | 0.057 | 0.223 | 6.500 (2, 102) | 0.113 | **0.002** | 2.750 (4, 102) | 0.097 | **0.032** |
| *Energy* | 2.786 (2, 51) | 0.099 | 0.071 | 5.714 (2, 102) | 0.101 | **0.004** | 4.021 (4, 102) | 0.136 | **0.005** |
| *Mental health* | 7.251 (2, 51) | 0.221 | **0.002** | 6.391 (2, 102) | 0.111 | **0.002** | 2.365 (4, 102) | 0.085 | 0.058 |
| *Social function* | 2.320 (2, 51) | 0.083 | 0.109 | 4.150 (2, 102) | 0.075 | **0.019** | 1.339 (4, 102) | 0.050 | 0.261 |
| *Pain* | 2.112 (2, 51) | 0.056 | 0.131 | 3.809 (2, 102) | 0.070 | **0.025** | 5.320 (4, 102) | 0.173 | **0.008** |
| *General health* | 8.128 (2, 51) | 0.242 | **< 0.001** | 8.626 (2, 102) | 0.145 | **< 0.001** | 5.166 (4, 102) | 0.168 | **0.008** |

Note: Results are presented as F- value, *η****²****:* Partial Eta Squared (Effect Size), P-value Significance *at p < 0.05*

Abbreviations: ABC = Active Balance Confidence Scale; DB = Diaphragmatic Breathing; DM = Diaphragmatic Mobilization; FSS = Fatigue Severity Scale; 5xSTS = Five Times Sit to Stand Test; mini-BEST = Mini Balance Evaluation System Test; SF-36 = Short Form -36; TUG = Timed Up & Go.
