# Supplementary material for: The effect of diaphragmatic breathing and diaphragmatic mobilization on physical performance, fear of falling, and quality of life in community-dwelling older adults: A randomized controlled trial
Source: PLoS One. 2026 Jan 5;21(1):e0339868. doi: 10.1371/journal.pone.0339868 (PMC12768353; doi:10.1371/journal.pone.0339868)
Supplement: S3 Table — The table presents the mean and standard deviation at baseline, post-treatment, and follow-up, and includes within-group change from baseline (Δ) with 95% confidence intervals (95% CI), where 95% CI are shown as lower and upper bound values. It also includes post hoc pairwise comparisons for both within-group and between-group differences. (DOCX) [file pone.0339868.s003.docx]

**S3 Table**

**Descriptive statistics of all outcome measures of all three intervention groups (N=54).**

|  |  | **Time** | | | | |
| --- | --- | --- | --- | --- | --- | --- |
| **Outcome Measures** | **Treatment Group** | **Baseline** | **Post-treatment** | **Follow-up** | **Δ Post-treatment** | **Δ Follow-up** |
| **mini-BEST (score)** | DB | 14.88 ± 1.52 | 15.27 ± 1.52 | 15.44 ± 1.46^*‡^ | 0.39 ± 0.92 (-0.07, 0.84) | 0.56 ± 0.86 (0.13, 0.98) |
|  | DB+DM | 15.61 ± 1.04 | 15.94 ± 1.11^‡^ | 16.11 ± 1.08^*‡^ | 0.33 ± 0.59 (0.04, 0.63) | 0.50 ± 0.71 (0.15, 0.85) |
|  | Control | ^1^5.00 ± 1.75 | 14.55 ± 1.54^*^ | 13.77 ±1.69^*†^ | -0.44 ± 0.62 (-0.75, -0.14) | -1.22 ± 1.06 (-1.75, -0.70) |
| **TUG (sec)** | DB | 16.91 ± 1.55 | 16.20 ± 1.22^*^ | 16.07 ± 1.28^*^ | -0.70 ± 0.94 (-1.17, -0.23) | -0.84 ± 0.96 (-1.32, -0.36) |
|  | DB+DM | 16.44 ± 1.48 | 15.64 ± 0.80^*‡^ | 15.39 ± 0.87^*†‡^ | -0.79 ± 1.11 (-1.35, -0.24) | -1.05 ± 1.24 (−1.67, −0.44) |
|  | Control | 18.04 ± 5.60 | 18.27 ± 5.46 | 18.47 ± 5.37^*^ | +0.23 ± 0.44 (0.01, 0.45) | +0.43 ± 0.60 (0.13, 0.73) |
| **Gait Velocity (m/sec)** | DB | 0.74 ± 0.10 | 1.02 ± 0.18^*‡^ | 1.04 ± 0.19^*‡^ | +0.27 ± 0.15 (0.20, 0.35) | +0.29 ± 0.18 (0.21, 0.38) |
|  | DB+DM | 0.73 ± 0.10 | 1.00 ± 0.15^*‡^ | 1.05 ± 0.15^*†‡^ | +0.28 ± 0.13 (0.22, 0.34) | +0.33 ± 0.15 (0.25, 0.40) |
|  | Control | 0.70 ± 0.08 | 0.64 ± 0.05^*^ | 0.64 ± 0.05^*^ | -0.06 ± 0.07 (-0.10, -0.03) | -0.06 ± 0.07 (-0.10, -0.03) |
| **5xSTS (sec)** | DB | 22.11 ± 1.84 | 21.94 ± 1.89 | 21.88 ± 1.93 | -0.17 ± 0.38 (-0.36, 0.02) | -0.22 ± 0.43 (-0.44, -0.01) |
|  | DB+DM | 22.22 ± 1.69 | 22.05 ± 1.86 | 21.94 ± 1.89 | -0.17 ± 0.38 (-0.36, 0.02) | -0.28 ± 0.46 (-0.51, -0.05) |
|  | Control | 21.39 ± 1.50 | 21.50 ± 1.50 | 21.61 ± 1.42 | +0.11 ± 0.32 (-0.05, 0.27) | +0.22 ± 0.43 (0.01, 0.44) |
| **ABC (%)** | DB | 62.94 ± 2.50 | 69.33 ± 3.83^*‡^ | 69.27 ± 3.65^*‡^ | 6.39 ± 2.95 (4.92, 7.86) | 6.33 ± 2.85 (4.92, 7.75) |
|  | DB+DM | 63.55 ± 2.61 | 71.27 ± 3.89^*‡^ | 71.33 ± 3.81^*‡^ | 7.72 ± 3.56 (5.95, 9.49) | 7.78 ± 3.66 (5.96, 9.60) |
|  | Control | 64.22 ± 0.80 | 62.22 ± 1.66^*^ | 61.83 ± 1.82^*†^ | -2.00 ± 1.78 (-2.89, -1.11) | -2.39 ± 1.97 (−3.37, -1.41) |
| **FSS (score)** | DB | 50.55 ± 4.20^‡^ | 45.16 ± 4.69^*‡^ | 45.00 ± 4.81^*‡^ | -5.39 ± 2.85 (-6.81, -3.97) | -5.55 ± 3.03 (-7.03, -4.08) |
|  | DB+DM | 49.94 ± 3.81^‡^ | 41.77 ± 4.64^*‡^ | 41.61 ± 4.96^*‡^ | -8.17 ± 4.91 (-10.61, -5.72) | -8.33 ± 5.122 (-10.88, -5.79) |
|  | Control | 54.33 ± 2.40 | 55.55 ± 2.09 | 55.55 ± 2.09 | +1.22 ± 1.16 (-0.64, 1.80) | +1.22 ± 1.16 (0.64, 1.16) |
| **SF-36 (score)** | | | | | | |
| *Physical function* | DB | 769.44 ± 42.49 | 783.33 ± 34.29^‡^ | 783.33± 34.29^‡^ | +13.89 ± 23.04 (2.43, 25.35) | +13.89 ± 23.04 (2.43, 25.35) |
|  | DB+DM | 755.55 ± 41.62 | 772.22 ± 39.19^*^ | 772.22 ± 39.19^*^ | +16.67 ± 24.25 (4.61, 28.73) | +16.67 ± 24.25 (4.61, 28.73) |
|  | Control | 752.78 ± 43.63 | 744.44 ± 37.92 | 744.44 ± 37.92 | -8.33 ± 19.17 (-17.87, 1.20) | -8.33 ± 19.17 (-17.87, 1.20) |
| *Role limits – Physical* | DB | 288.89 ± 75.84 | 311.11 ± 83.23 | 311.11 ± 83.23 | +22.22 ± 42.78 (0.95, 43.50) | +22.22 ± 42.78 (0.95, 43.50) |
|  | DB+DM | 305.56 ± 72.53 | 327.78 ± 57.45^‡^ | 333.33 ± 59.41^‡^ | +22.22 ± 42.78 (0.95, 43.50) | +27.78 ± 46.09 (4.86, 50.70) |
|  | Control | 266.67 ± 48.51 | 266.67 ± 48.51 | 266.67 ± 48.51 | 0.00 ± 0.00 (0.00, 0.00) | 0.00 ± 0.00 (0.00, 0.00) |
| *Role limits – Emotional* | DB | 283.33 ± 38.35 | 288.89 ± 32.34 | 294.44 ± 23.57 | +5.56 ± 23.57 (-6.17, 17.28) | +11.11 ± 32.34 (-4.97, 27.19) |
|  | DB+DM | 261.11 ± 50.16 | 283.33 ± 38.35 | 288.89 ± 32.34 | +22.22 ± 42.78 (0.95, 43.50) | +27.78 ± 46.09 (4.86, 50.70) |
|  | Control | 266.67 ± 48.51 | 266.67 ± 48.51 | 266.67 ± 48.51 | 0.00 ± 0.00 (0.00, 0.00) | 0.00 ± 0.00 (0.00, 0.00) |
| *Energy* | DB | 273.33 ± 21.69 | 280.00 ± 20.58 | 280.00 ± 20.58 | +6.67 ± 11.88 (0.76, 12.58) | +6.67 ± 11.88 (0.76, 12.58) |
|  | DB+DM | 276.67 ± 20.86 | 282.22 ± 18.00^‡^ | 282.22 ± 18.00^‡^ | +5.56 ± 11.49 (-0.16, 11.27) | +5.56 ± 11.49 (-0.16, 11.27) |
|  | Control | 267.78 ± 20.74 | 265.55 ± 17.89 | 265.55 ± 17.89 | -2.22 ± 6.47 (-5.44, 0.99) | -2.22 ± 6.47 (-5.44, 0.99) |
| *Mental health* | DB | 433.33 ± 24.73^‡^ | 436.67 ± 19.70^‡^ | 436.67 ± 19.70^‡^ | +3.33 ± 15.72 (-4.48, 11.15) | +3.33 ± 15.72 (-4.48, 11.15) |
|  | DB+DM | 428.89 ± 21.93 | 436.67 ± 19.70^*‡^ | 436.67 ± 19.70^*‡^ | +7.78 ± 10.03 (2.79, 12.77) | +7.78 ± 10.03 (2.79, 12.77) |
|  | Control | 415.55 ± 10.97 | 415.55 ± 10.97 | 415.55 ± 10.97 | 0.00 ± 0.00 (0.00, 0.00) | 0.00 ± 0.00 (0.00, 0.00) |
| *Social function* | DB | 191.67 ± 12.13 | 194.44 ± 10.69 | 194.44 ± 10.69 | +2.78 ± 11.79 (-3.08, 8.64) | +2.78 ± 11.79 (-3.08, 8.64) |
|  | DB+DM | 190.28 ± 12.54 | 194.44 ± 10.69 | 195.83 ± 9.59 | +4.17 ± 9.59 (-0.60, 8.93) | +5.56 ± 10.69 (0.24, 10.87) |
|  | Control | 186.11 ± 15.39 | 186.11 ± 15.39 | 186.11 ± 15.39 | 0.00 ± 0.00 (0.00, 0.00) | 0.00 ± 0.00 (0.00, 0.00) |
| *Pain* | DB | 164.17 ± 12.86 | 166.39 ± 9.97 | 166.39 ± 9.97 | +2.22 ± 6.00 (-0.76, 5.20) | +2.22 ± 6.00 (-0.76, 5.20) |
|  | DB+DM | 161.39 ± 17.72 | 167.50 ± 12.86^*‡^ | 167.50 ± 12.86^*‡^ | +6.11 ± 9.93 (1.17, 11.05) | +6.11 ± 9.93 (1.17, 11.05) |
|  | Control | 159.17 ± 16.38 | 156.94 ± 14.57 | 156.94 ± 14.57 | -2.22 ± 6.47 (-5.44, 0.99) | -2.22 ± 6.47 (-5.44, 0.99) |
| *General health* | DB | 394.44 ± 35.93 | 406.94 ± 35.15^‡^ | 408.33 ± 34.29^*‡^ | +12.50 ± 21.44 (1.84, 23.16) | +13.89 ± 21.39 (3.25, 24.53) |
|  | DB+DM | 409.72 ± 42.99^‡^ | 423.61 ± 35.84^‡^ | 423.61 ± 35.84^‡^ | +13.89 ± 23.04 (2.43, 25.35) | +13.89 ± 23.04 (2.43, 25.35) |
|  | Control | 379.17 ± 26.08 | 375.00 ± 24.25 | 375.00 ± 24.25 | -4.17 ± 9.59 (-8.93, 0.60) | -4.17 ± 9.59 (-8.93, 0.60) |

Note: Data are presented as mean ± SD

Δ indicates change from baseline and is presented as mean ± SD with 95% Confidence interval (95% CI), where 95% CI are shown as lower and upper bound values.

^*^*p < 0.05*, significantly different from baseline

^†^*p < 0.05*, significantly different from post-treatment

^‡^*p < 0.05*, significantly different from the control group

Abbreviations: ABC = Active Balance Confidence Scale; DB = Diaphragmatic Breathing; DM = Diaphragmatic Mobilization; FSS = Fatigue Severity Scale; 5xSTS = Five Times Sit to Stand Test; mini-BEST = Mini Balance Evaluation System Test; SF-36 = Short Form -36; TUG = Timed Up & Go.
