# Supplementary material for: The effect of diaphragmatic breathing and diaphragmatic mobilization on physical performance, fear of falling, and quality of life in community-dwelling older adults: A randomized controlled trial
Source: PLoS One. 2026 Jan 5;21(1):e0339868. doi: 10.1371/journal.pone.0339868 (PMC12768353; doi:10.1371/journal.pone.0339868)
Supplement: S1 File — The approved trial study protocol outlining the study design, participants, interventions, outcome measures, and ethical approval obtained from the Ethics Committee of Chulalongkorn University. (DOCX) [file pone.0339868.s004.docx]

**The effect of diaphragmatic breathing and diaphragmatic mobilization on physical performance, fear of falling, and quality of life in older adults: a randomized controlled trial**

**Treatment protocol details**

Experienced healthcare professionals, including physiotherapists who will not be blind to the study protocols, will conduct the screening tests. These assessments will be organized in clinical settings equipped with the necessary facilities. The procedures will occur during scheduled appointments, ensuring efficient use of time and resources. To prevent falls during screening, ensure a hazard-free environment, provide supervision and assistance as needed, and educate participants on cautious movement. Comprehensive explanations about the project's objectives, procedures, potential risks, benefits, and participants' rights will be provided. Following this, explicit consent will be obtained from each participant before their involvement in the research.

All participants' physical information, including height, weight, and body mass index, will be kept on file [1]. All selected tests are crucial for a comprehensive evaluation, each serving a specific purpose in assessing different aspects of participants' health and well-being. These assessments are minimally invasive, require minimal time commitment, and are essential for ensuring the effectiveness and safety of the intervention. To select the important tests, subjects will be recruited based on the mini-BEST score of less than 17, having disturbed balance and chances to fall as indicated by their breathing pattern. However, these subjects are ambulatory and living in a community without using any assistive device. After the screening examination, subjects will be randomized into one of the three groups (Group 1: Diaphragmatic Breathing, Group 2: Diaphragmatic Breathing and Diaphragmatic Mobilization, Group 3: Control (subjects will be in waiting list).

In the study, blinding will be maintained as a single-blinded protocol where only the outcome assessors will be blinded to the group assignments. Randomization will be conducted by an independent researcher not involved in participant assessment or recruitment. This external individual will generate the random allocation sequence using a validated random number generator or software. The group allocation details will be enclosed in opaque, sealed envelopes or stored in a secure, password-protected electronic system, ensuring concealment of allocation.

To maintain the single-blinded nature of the study, the assessors responsible for outcome evaluations will not know the group assignments. This blinding process aims to prevent assessment bias. All other study participants, including the subjects and those involved in delivering interventions, will be aware of the group allocation due to the nature of the interventions. These measures align with the single-blinded approach recommended for the research design and ensure an unbiased assessment of outcomes.

For each group all the variables will be measured, balance + gait velocity, lower extremity strength, fear of fall, fatigue, and quality of life with the outcome measuring tools, mini-BESTest, 5 times Sit to stand (5XSTS), Assessment of balance confidence (ABC), fatigue severity scale (FSS), Short form 36 (short-SF 36) respectively. After measuring all the variables, techniques for the respective groups will be applied: Group 1: Diaphragmatic breathing, Group 2: Diaphragmatic breathing and diaphragmatic mobilization, Group 3: Control (subjects will be on waiting list).

To give the interventions to the respective group, physiotherapists having a minimum of 5 years of experience will be selected under the supervision of senior physiotherapist [2]. The senior physiotherapist responsible for selecting physiotherapists for diaphragmatic breathing and diaphragmatic mobilization interventions will be the one having more than 10 years of experience in cardiopulmonary physical therapy, designated as the lead clinician overseeing personnel assignments for these specialized roles. Research assistants (Physiotherapists) will receive thorough training on protocol implementation to ensure adherence to study procedures. They will provide opportunities for clarification or additional support as needed.

For group 2, participants had diaphragmatic mobilization along with diaphragmatic breathing. Given the nature of the technique and the absence of extensive physical touch, participants' preference for the gender of the physiotherapist might not be a primary concern. Moreover, consent will be sought from participants before any intervention, ensuring their comfort and willingness to engage in the process. This oversight will help maintain consistency and compliance with the established protocols throughout the research project.

Training sessions will be conducted individually to ensure personalized attention and effective management of participant schedules. Clean and suitable spaces, such as physiotherapy clinics or designated areas within healthcare facilities, will be utilized for training sessions, where participants can lay down comfortably. Participants will arrange their transportation to the training location. A structured orientation session will be conducted for physiotherapists to familiarize them with the research project, ensuring standardized performance. This session will cover study objectives, protocols, and expectations, fostering consistency in intervention delivery. Treatment duration will be 8 weeks, 2 times per week (total 16 sessions).

Total Treatment time will be:

Group 1: diaphragmatic breathing for a total of 20 minutes.

Group 2: diaphragmatic breathing + diaphragmatic mobilization for a total of 20 minutes.

Group 3: Subjects will be on the waiting list (no time will be spent with them).

Measurements will be taken at:

1. Pre-treatment (pre-test)
2. Immediate post-treatment (8^th^ week of treatment)
3. Post-treatment (10^th^ week).

Participants will be strongly encouraged to attend all scheduled training sessions, with provisions made to facilitate attendance, such as providing transportation assistance and ensuring accessibility to the training location. Efforts will be made to minimize barriers to participation and maximize adherence to the intervention program. Participants will be allowed to miss up to two sessions without being excluded from the study. If a participant misses a session, they will be provided with the opportunity to make up for the missed session at a later date, if feasible. They will be provided with detailed instructions on how to prepare for training, including guidelines on appropriate attire.

Here are some guidelines:

1. Dress comfortably in breathable clothing suitable for physical activity.
2. Avoid loose or restrictive clothing that may impede movement.
3. Bring a water bottle to stay hydrated during training sessions.
4. Remove any jewelry or accessories that could pose a safety hazard.
5. Follow any additional instructions provided by the research team regarding attire or preparation for specific training activities.

This information will be communicated clearly to ensure participants arrive adequately prepared for the training sessions.

Some safety guidelines will be ensured, such as:

1. Keep pathways clear and well-lit.
2. Install handrails and grab bars in critical areas.
3. Use non-slip mats.
4. Perform exercises and assessments in a safe environment with complete supervision.

In case a fall happens, a fall response plan will be utilized, including immediate assistance and rehabilitation.

**Treatment details**

**Diaphragmatic breathing**

Diaphragmatic breathing exercise will be performed as the patient places one hand on the chest and the other hand on the abdomen with minimal chest movement, breathing slowly and deeply through the nose for four seconds and exhaling through the mouth for six seconds approximately [3]. The patient will be instructed not to move the hand on the chest and push the air to the abdomen, causing the hand there to move [3]. Normal resting respiration involves approximately 10–12 breaths/min [4]. When asked to breathe deeply, participants should be able to slow this rate and take 10 seconds to complete a cycle of inhalation and exhalation, about six breaths/min [5]. Blood pressure will be monitored before and at the end of the intervention.

**Treatment details for group 1**

| Duration | 8 weeks [6] |
| --- | --- |
| Intensity | 2 times/ week (2*8=16) |
| Length of session | 3 sets of total 20 minutes [7] |
| Repetitions per set | 6 min breathing (6 breaths/min) = 36 breaths/set [5] |
| Sequence | 6 min breathing each set with 1 min interval |

Blood pressure will be monitored before and after each session in both intervention groups (Group 1: DB and Group 2: DB+DM). To monitor the efficacy of the 1-minute rest interval between sets, participants will be queried regarding their perceived exertion using a standardized Perceived Exertion Rating Scale. This approach ensures a comprehensive assessment of subjective exertion levels, aiding in determining the appropriateness of the prescribed rest duration in the exercise protocol.

**Borg's rate of perceived exertion (RPE)**

Various studies have used the RPE scale to monitor the intensity of exercise and to know the exertion level after diaphragmatic breathing exercises [8-10].

Initial Assessment:

- - Before initiating the exercise, the patient is instructed to rate their perceived exertion using the Borg RPE scale, which typically ranges from 6 to 20, with 6 indicating no exertion at all and 20 indicating maximal exertion.
  - The patient's initial RPE provides a baseline measure of their perceived exertion level at the beginning of the exercise session.

During Exercise:

- - Throughout the exercise session, (after 6 min each) the patient continuously monitors their perceived exertion level using the Borg RPE scale (After session 1, 2 and 3).
  - The acceptable range of RPE to sustain the exercise should fall within the specified range of 11 to 13 on the Borg scale [10]. This range indicates a moderate to somewhat hard level of exertion.

End of Exercise:

- - At the conclusion of the prescribed exercise duration, typically after 20 minutes, the patient records their final RPE value. This value reflects their perceived exertion level at the end of the exercise bout.

Rest Period:

- - Following the exercise period, a one-minute rest period is initiated.
  - During this rest period, the patient's RPE is monitored to ensure it falls within the acceptable range of 11 to 13 on the Borg scale [10].
  - If the RPE falls within this range, the patient proceeds to the next set of exercises.
  - If the RPE is outside the acceptable range, an additional minute of rest may be provided, and the patient's RPE is reassessed until it falls within the acceptable range.
  - This process ensures that the patient adequately recovers between exercise sets and maintains an appropriate level of exertion throughout the session.

**Progression protocol**

Progressive overload and systematic escalation of the stress imposed on the muscles during training are essential to promote ongoing muscle adaptations [11, 12]. Progression is instrumental in eliciting neural and intramuscular adaptations, enabling individuals to endure higher training volumes, and enhancing the efficacy of training over an extended period [13-15].

Previous studies [7, 16, 17] have used the following sequential progression to promote postural stability via the diaphragmatic breathing exercise:

a) Supine breathing & Crocodile breathing.

b) Supine breathing with TheraBand & Crocodile breathing with TheraBand

c) Seated breathing & 90/90/90 breathing; and

d) Seated breathing with TheraBand & 90/90/90 breathing with TheraBand.

**Progression details**

- Progression 1: (First & second week) Supine breathing & Crocodile breathing [7, 16].
- Progression 2: (Third & fourth week) Supine breathing with TheraBand & Crocodile breathing with TheraBand [7, 16].
- Progression 3: (Fifth & sixth week) Seated breathing & 90/90/90 breathing [7, 16]*.*
- Progression 4: (Seventh & eighth week) Seated breathing with TheraBand & 90/90/90 breathing with TheraBand [7, 16].

**TheraBand**

For the resistance purpose, a yellow Thera-Band^®^ with a resistance of 1.3 kg will be used [18]. The same resistance will be used for every position.

**Details of diaphragmatic breathing in different positions**

1. **Supine Breathing:**

The supine position may place the diaphragm at a mechanical disadvantage without the optimal influence of gravity to assist with caudal descent [19] [20]. It is also possible that the degree of pelvic tilt or position of the upper extremities may affect DB efforts. For example, a posterior pelvic tilt may facilitate DB. In contrast, internal rotation and adduction of the upper extremities may inhibit upper chest wall motion [21] [19].

Procedure: Participants will be instructed to assume a hook-lying position with their arms positioned comfortably. They will be guided to focus on diaphragmatic breathing, ensuring that their breath reaches their lower abdomen and posterior of the chest wall. Emphasis will be placed on maintaining depressed ribs and keeping the shoulders and neck relaxed [7, 16].

For the resistance component of the exercise, a TheraBand will be added around the thoracolumbar junction [7, 16].

1. **Crocodile breathing**

Crocodile breathing entails engaging the diaphragm while in a prone position, enhancing pelvic control, promoting correct sequencing, and activating core muscles. This leads to stabilizing the shoulder girdle, enabling expansion of the lower rib cage [22]. Crocodile breathing mainly involves breathing by diaphragmatic activation. When one uses their diaphragm to breathe instead of the more typical “shallow breathing” of the intercostals muscles, greater thoracic mobility is established, and enhanced core muscle activation is enabled [22].

Procedure: Participants will be directed to lie prone with their hands forming a diamond shape to support their forehead. They will be instructed to push their ribs out laterally and breathe deeply down to the sacrum [7, 16].

For the resistance component of training, a TheraBand will be positioned beneath the participant's thoracolumbar junction [7, 16].

1. **Seated breathing**

In an erect posture like sitting, the diaphragm moves downward due to reduced pressure from the abdominal contents, resulting in an anticipated decrease in its excursion [23]. The seated position relieves the diaphragm from the burden of intra-abdominal contents, assisting its piston-like downward displacement ability. Additionally, it prevents the gravitational collapse of the small airways in the posterior, dependent lung zones [24]. Individuals frequently initiate stabilization exercises in a supine position but advance to more functional upright postures as their performance improves [25].

Procedure: Participants will be seated on a firm surface with their knees, hips, and ankles all forming 90-degree angles. They will be instructed to sit in an upright manner, imagining a "string pulling them up from the top of their head," while adhering to the previously mentioned breathing instructions: avoiding lower rib flare, breathing deeply, and keeping their shoulders, neck, and arms relaxed [7, 16].

For the resistance component of training, a TheraBand will be positioned beneath the participant's thoracolumbar junction [7, 16].

1. **90/90/90 Breathing:**

The body's positioning with the hip and knee flexed at a 90-degree angle induces relative flexion in the lumbar spine, a posterior pelvic tilt, and internal rotation of the ribs. This configuration aims to optimize the Zone of Apposition (ZOA) [26]. The Zone of Apposition (ZOA) refers to the section of the diaphragm that includes the cylindrical portion, resembling a dome or umbrella (Hodges et al., 1997). Significantly, the ZOA is under the influence of the abdominal muscles, playing a crucial role in regulating diaphragmatic tension [27].

Procedure: Participants will be positioned in the 90/90/90 configuration and will be instructed to hold their legs while adhering to the previously mentioned breathing instructions, managing their ribs and thoracolumbar junction, breathing deeply, and keeping their shoulders, neck, and arms relaxed [7, 16].

For the resistance component of training, a **TheraBand** will be positioned beneath the participant’s thoracolumbar junction [7, 16].

**Diaphragmatic Mobilization**

The participants will lie on their backs with their limbs relaxed. For the diaphragmatic mobilization technique, the breathing pattern will follow the same parameters established for diaphragmatic breathing: a structured pattern of inhalation for 4 seconds and exhalation over 6 seconds [3].

Diaphragmatic mobilization will be applied to the mobilization group by the physiotherapist while the participant will be in a supine and relaxed position. Thumbs will be placed on the xiphoid process. The costae will be grasped and closed together, with the remaining fingers and the fingertips reaching into the lumbar region and drawing a virtual 8 motion. For a total of 3 sessions, this technique will be applied in each session for 3 minutes with a brief rest period between each set to help the participant relax and improve their breathing.


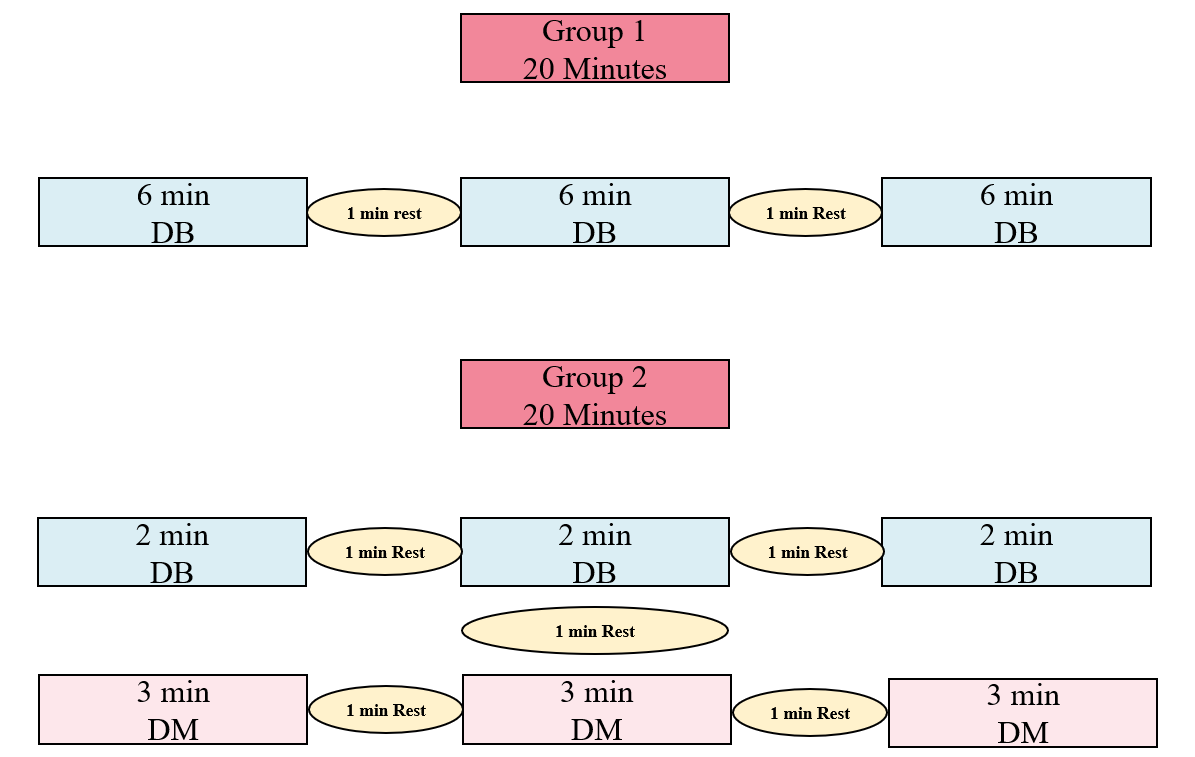


**Treatment Protocol for Group 1 & Group 2**

**DB; Diaphragmatic breathing, DM; Diaphragmatic mobilization**

1. Evans ES, Ketcham CJ, Hibberd JC, Cullen ME, Basiliere JG, Murphy DL. Examination of clinical and laboratory measures of static and dynamic balance in breast cancer survivors. Physiotherapy Theory and Practice. 2021;37(11):1199-209. doi: 10.1080/09593985.2019.1692391.

2. Kim S-H, Shin H-J, Cho H-Y. Impact of Types of Breathing on Static Balance Ability in Healthy Adults. International journal of environmental research and public health. 2022;19(3):1205.

3. Şahin O, Kocamaz D. Effects of diaphragmatic mobilization and diaphragmatic breathing exercises on pain and quality of life in individuals with shoulder pain: a randomized controlled trial. International Journal of Disabilities Sports and Health Sciences. 2021;4(2):113-23.

4. Courtney R. The functions of breathing and its dysfunctions and their relationship to breathing therapy. International Journal of Osteopathic Medicine. 2009;12(3):78-85.

5. Nelson N. Diaphragmatic Breathing: The Foundation of Core Stability. Strength & Conditioning Journal. 2012;34(5):34-40. doi: 10.1519/SSC.0b013e31826ddc07. PubMed PMID: 00126548-201210000-00005.

6. Stephens RJ, Haas M, Moore III WL, Emmil JR, Sipress JA, Williams A. Effects of diaphragmatic breathing patterns on balance: a preliminary clinical trial. Journal of manipulative and physiological therapeutics. 2017;40(3):169-75.

7. Stephens RJ, Haas M, Moore WL, Emmil JR, Sipress JA, Williams A. Effects of Diaphragmatic Breathing Patterns on Balance: A Preliminary Clinical Trial. Journal of Manipulative and Physiological Therapeutics. 2017;40(3):169-75. doi: <https://doi.org/10.1016/j.jmpt.2017.01.005>.

8. Kim C-W, Lee D-Y, Hong J-H, Yu J-H, Kim J-S. Initial Effects of Different Recovery Methods of Vital Sign After. Annals of the Romanian Society for Cell Biology. 2021:1489-99.

9. Shukla M, Chauhan D, Raj R. Breathing exercises and pranayamas to decrease perceived exertion during breath-holding while locked-down due to COVID-19 online randomized study. Complementary Therapies in Clinical Practice. 2020;41:101248. doi: <https://doi.org/10.1016/j.ctcp.2020.101248>.

10. Lee H-Y, Cheon S-H, Yong M-S. Effect of diaphragm breathing exercise applied on the basis of overload principle. Journal of physical therapy science. 2017;29(6):1054-6.

11. Kraemer WJ, Ratamess NA. Fundamentals of resistance training: progression and exercise prescription. Medicine & science in sports & exercise. 2004;36(4):674-88.

12. Medicine ACoS. American College of Sports Medicine position stand. Progression models in resistance training for healthy adults. Medicine and science in sports and exercise. 2009;41(3):687-708.

13. Sale DG. Neural adaptation to resistance training. Medicine and science in sports and exercise. 1988;20(5 Suppl):S135-45.

14. Peterson MD, Rhea MR, Alvar BA. Maximizing strength development in athletes: a meta-analysis to determine the dose-response relationship. The Journal of Strength & Conditioning Research. 2004;18(2):377-82.

15. Škarabot J, Brownstein CG, Casolo A, Del Vecchio A, Ansdell P. The knowns and unknowns of neural adaptations to resistance training. European Journal of Applied Physiology. 2021;121:675-85.

16. Otadi K, Nakhostin Ansari N, Sharify S, Fakhari Z, Sarafraz H, Aria A, Rasouli O. Effects of combining diaphragm training with electrical stimulation on pain, function, and balance in athletes with chronic low back pain: a randomized clinical trial. BMC Sports Science, Medicine and Rehabilitation. 2021;13(1):20. doi: 10.1186/s13102-021-00250-y.

17. Gaffar AF, Waghray S, Balne NK. Efficacy of Balance Scales in Fall Risk Identification in Elderly with Breathing Pattern Dysfunction-An Observational Study. International Journal of Health Sciences and Research. 2019;9(2):101-7.

18. Karaket S, andDonnapa Chaisombut SP. Positive Effects of Group Exercises Using a Resistance Band on Trunk Balance of Elderly Thais in Rural Communities.

19. Willeput R, Vachaudez J, Lenders D, Nys A, Knoops T, Sergysels R. Thoracoabdominal motion during chest physiotherapy in patients affected by chronic obstructive lung disease. Respiration. 1983;44(3):204-14.

20. Sackner MA, Gonzalez H, Rodriguez M, Belsito A, Sackner DR, Grenvik S. Assessment of asynchronous and paradoxic motion between rib cage and abdomen in normal subjects and in patients with chronic obstructive pulmonary disease. American Review of Respiratory Disease. 1984;130(4):588-93.

21. Massery M, Frownfelter D. Facilitating ventilatory patterns and breathing strategies. Principles and Practice of Cardiopulmonary Physical Therapy, 3rd St Louis: Mosby-YearBook. 1996:383-416.

22. Verma CV, Jere GS, Sheth RD, Bharmal RN. Effectiveness of crocodile breathing versus prone position in patients with COVID-19: A pilot study. Indian Journal of Respiratory Care. 2022;11(3):219-23.

23. Gerscovich EO, Cronan M, McGahan JP, Jain K, Jones CD, McDonald C. Ultrasonographic evaluation of diaphragmatic motion. Journal of ultrasound in medicine. 2001;20(6):597-604.

24. Lemyze M, Mallat J, Duhamel A, Pepy F, Gasan G, Barrailler S, et al. Effects of Sitting Position and Applied Positive End-Expiratory Pressure on Respiratory Mechanics of Critically Ill Obese Patients Receiving Mechanical Ventilation*. Critical Care Medicine. 2013;41(11).

25. Hellyer NJ, Andreas NM, Bernstetter AS, Cieslak KR, Donahue GF, Steiner EA, et al. Comparison of Diaphragm Thickness Measurements Among Postures Via Ultrasound Imaging. PM&R. 2017;9(1):21-5. doi: <https://doi.org/10.1016/j.pmrj.2016.06.001>.

26. Mahendran M. Effectiveness of 90/90 Supported Hip Shift with Hemi Bridge Ball and Balloon Exercises along with Forced Breathing Technique on Improving Pulmonary Function and Reducing Pain in Patients with Chronic Back Pain: Nandha College of Physiotherapy, Erode; 2019.

27. Hodges P, Butler J, McKenzie D, Gandevia S. Contraction of the human diaphragm during rapid postural adjustments. The Journal of physiology. 1997;505(Pt 2):539.
