# Supplementary material for: The effect of diaphragmatic breathing and diaphragmatic mobilization on physical performance, fear of falling, and quality of life in community-dwelling older adults: A randomized controlled trial
Source: PLoS One. 2026 Jan 5;21(1):e0339868. doi: 10.1371/journal.pone.0339868 (PMC12768353; doi:10.1371/journal.pone.0339868)
Supplement: Attachment — Submitted filename: New Microsoft Word Document.docx [file pone.0339868.s006.docx]

1. Line number 315, 346, 363: Avoid the term elderly since it is considered as ageist. Older adults are a more acceptable term and I suggest to use the term older adults uniformly throughout the manuscript
2. Good study and well reported
3. Reference 58 and 59 are superscripted. Follow the journal guidelines closely.
