# Supplementary material for: The effect of diaphragmatic breathing and diaphragmatic mobilization on physical performance, fear of falling, and quality of life in community-dwelling older adults: A randomized controlled trial
Source: PLoS One. 2026 Jan 5;21(1):e0339868. doi: 10.1371/journal.pone.0339868 (PMC12768353; doi:10.1371/journal.pone.0339868)
Supplement: Attachment — Submitted filename: Response to Reviewers revised 12-07-25.docx [file pone.0339868.s007.docx]

**Manuscript Title:** The Effect of Diaphragmatic Breathing and Diaphragmatic Mobilization on Physical Performance, Fear of Falling, and Quality of Life in Older Adults
**Manuscript ID:** PONE-D-25-10162
**Corresponding Author:** Associate Professor Anong Tantisuwat

**Date:** 12 July 2025

Dear Dr. Miray Budak and Reviewers,

Thank you for your constructive feedback and the opportunity to revise and improve our manuscript. We have thoroughly addressed all the comments from the editorial office and reviewers. Below is a point-by-point table listing each comment along with our response. All revisions are marked in the file titled “Revised Manuscript with Track Changes.” We hope these revisions meet your expectations, and we sincerely appreciate the time you took to provide us with your valuable feedback.

| **Comments from the Reviewers** | **Responses to the Reviewers** |
| --- | --- |
| **Reviewer 1** |  |
| 1. Line 128 – Is the effect size of 0.3 standardized? If not, what SD was used? | We appreciate your question and confirm that the effect size of **0.3** used in our sample size calculation was a **standardized effect size (Cohen’s f)**, derived from our pilot study.  We have now clarified this information in the revised manuscript on page 6, lines 128, under the heading **“Sample size and randomization”** |
| 2. Line 130 – Clarify whether the calculated sample size (n=39) is total or per group. Describe the test used in G*Power. | We value your comment. The calculated sample size of **39 refers to the total number of participants**, not per group. This number was derived from the pilot data of our study and includes an adjustment for an anticipated 30% attrition rate.  The sample size was calculated using **G*Power (version 3.1.9.4)** with the following input parameters:   - Effect size (*f*) = 0.3 - α = 0.05 - Power (1 – β) = 0.80 - Number of groups = 3 - Number of measurements = 3   We have now clarified in the revised manuscript, under the heading **“Sample Size and Randomization”** (page 6, line 128), that the test used in G*Power was **Repeated Measures ANOVA (within–between interaction)**. |
| 3. Figure 1 – Dropout inconsistency: each group lost 2 participants, yet shows 18 analyzed. | Thank you for pointing out the inconsistency in Figure 1. We have revised Figure 1 to clearly distinguish between participants who completed the study and those included in the analysis. Each group now shows “Completed the study (n = 16)” followed by “Analyzed (n = 18; intention-to-treat)” in the final row, to transparently reflect participant attrition and the analysis approach.  Furthermore, this clarification has been incorporated into the manuscript and is highlighted in the “manuscript with tracked changes” under the **Statistical Analysis section** (page 9, line 192):  “An intention-to-treat (ITT) approach was used, including the data of all 54 randomized participants (n = 18 per group) in the analysis. Although 6 participants (2 per group) did not complete the study, their missing data were managed using the last observation carried forward (LOCF) method.  In addition, the caption for Figure 1 has been revised accordingly and is highlighted in the **Supporting Information section** (page 23, line 603). |
| **Reviewer 2** |  |
| Why did you not present results by charts and diagrams? | Thank you for your thoughtful question. We value visual data presentation and agree that charts and diagrams help highlight strong trends or clinically meaningful effects. In our study, although several outcomes reached statistical significance, their effect sizes are relatively small and do not consistently exceed the minimal clinically important difference (MCID) or minimal detectable change (MDC) thresholds. As a result, the clinical relevance of the findings remains modest.  In light of this context, a table-based presentation was considered the most appropriate and transparent method to illustrate nuanced group differences and small changes over time, thereby avoiding the accentuation of effects that may lack clinical significance. This methodology enables readers to assess the data independently and comprehensively, thereby assisting in preventing overinterpretation of the results.  Furthermore, the full results are provided in S1–S3 Tables, including effect sizes (η²), *p*-values, and descriptive statistics across all time points, offering a comprehensive and objective view of the findings. |
| **Reviewer 3** |  |
| 1. Avoid the term elderly since it is considered as ageist. Older adults are a more acceptable term and I suggest to use the term older adults uniformly throughout the manuscript. | Dear reviewer, thank you for your concern. We fully acknowledge the importance of using respectful and first-person language. Throughout the manuscript, we have consistently used the term “older adults” instead of “elderly.” In the few instances where “elderly” appeared, it was part of the original terminology used in cited studies. In those cases, we have revised the language to “older adults” while still accurately representing the source. We have carefully reviewed and updated the revised manuscript to ensure consistency with the preferred terminology. |
| 2. “Good study and well reported.” | We appreciate your kind and encouraging feedback. |
| 3. References 58 and 59 are superscripted. | Dear reviewer, we have corrected the formatting of References **58 and 59** to align with the journal’s reference style guidelines. |
| **Editor comments** |  |
| 1. Please ensure your manuscript meets PLOS ONE’s formatting requirements. | Revised manuscript has been updated according to the formatting requirements of the PLOS ONE. |
| 2. Competing Interests should state “The authors have declared that no competing interests exist.” In the online submission form. | Competing interest statements have been updated in the online submission system as “The authors have declared that no competing interests exist”. |
| 3. Please provide a complete Data Availability Statement in the submission form. | Data availability statement has been written in the submission form as “All data are in the manuscript and/or supporting information files” |
| 4. Amend submission data to include author Tsuyoshi Asai. | The authorship list has been updated in the revised manuscript and the submission data. |
| 5. Amend the authorship list to include Muhammad Awais Tassawar. | The authorship list has been updated in the revised manuscript. |
| 6. Upload figures on the Preflight Analysis and Conversion Engine (PACE) digital diagnostic tool, while resubmission. | Figures are uploaded as supporting information files separately; therefore, it is not required this step. |

Regarding the suggestion to publish the protocol separately on protocols.io, the intervention procedures are already comprehensively described in the Methods section and visually illustrated in Supporting Figures 2 and 3, offering sufficient clarity and reproducibility for readers. Therefore, we have opted not to publish the protocol separately on protocols.io for this study. We remain open to doing so in future research involving more complex or multi-phase intervention protocols.

Kind regards,
Anong Tantisuwat, PT, PhD, MPA

Associate Professor

Deputy Dean for Academic Affairs and Education, Information Technology, Public Relations, and Corporate Image

Mobile: +6663 5856363

E-mail: [anong.ta@chula.ac.th](mailto:anong.ta@chula.ac.th), [anongt@gmail.com](mailto:anongt@gmail.com)
