# Supplementary material for: The effect of diaphragmatic breathing and diaphragmatic mobilization on physical performance, fear of falling, and quality of life in community-dwelling older adults: A randomized controlled trial
Source: PLoS One. 2026 Jan 5;21(1):e0339868. doi: 10.1371/journal.pone.0339868 (PMC12768353; doi:10.1371/journal.pone.0339868)
Supplement: Attachment — Submitted filename: Response to Reviewers.docx [file pone.0339868.s009.docx]

**Manuscript Title:** The Effect of Diaphragmatic Breathing and Diaphragmatic Mobilization on Physical Performance, Fear of Falling, and Quality of Life in Older Adults
**Manuscript ID:** PONE-D-25-10162
**Corresponding Author:** Associate Professor Anong Tantisuwat

**Date:** 01 Dec 2025

Dear Dr. Miray Budak and Reviewers,

Thank you for your constructive feedback and the opportunity to revise and improve our manuscript. We have thoroughly addressed all the comments from the reviewer. Below is a point-by-point table listing each comment along with our response. All revisions are marked in the file titled “Revised Manuscript with Track Changes.” We hope these revisions meet your expectations, and we sincerely appreciate the time you took to provide us with your valuable feedback.

| **Comments from the Reviewer** | **Responses to the Reviewers** |
| --- | --- |
| **Reviewer 4** |  |
| **Title**   - The title could be more precise and should indicate that the study focuses on community-dwelling older adults. | We sincerely thank the reviewer for this helpful suggestion. To improve the precision and clearly reflect the target population, we have revised the title as recommended. The updated title is:  “The Effect of Diaphragmatic Breathing and Diaphragmatic Mobilization on Physical Performance, Fear of Falling, and Quality of Life in Community-Dwelling Older Adults: A Randomized Controlled Trial.” (Page 1, lines 1-3) |
| **Abstract (lines 22–43)**   - Lines 33–40: Effect sizes for significant results are not reported. - Lines 26, 40: The difference between the DB and DB+DM groups is unclear. - Lines 41–42: The conclusion appears overly optimistic despite no improvement in post-treatment balance. | We sincerely thank the reviewer for these thoughtful and constructive comments. We have revised the abstract to enhance clarity, transparency, and alignment with our findings. The key revisions are as follows:   - We have now included effect sizes (partial eta squared) for all significant group-by-time interactions to clearly communicate the magnitude of the observed effects (Abstract; page 2, lines 32–40). - To improve understanding of the intervention structure, we have clarified the group labels, explicitly indicating that the second group received an additive intervention combining Diaphragmatic Breathing with Diaphragmatic Mobilization (Abstract; page 2, lines 27–28). - We have revised the conclusion to ensure it reflects the results more precisely, particularly acknowledging the limited change in post-treatment balance, and have adjusted the tone to avoid over-interpretation of the outcomes (Abstract; page 3, lines 41–43). |
| **Introduction (lines 46–106)**   - Lines 82–88: The distinct mechanism of DM compared to DB is not explained. - Lines 74–78, 85–88: References to studies in younger adults are cited without caution regarding generalizability to older adults. - Lines 101–104: The hypothesis is overly broad and lacks prioritization. - The section is lengthy and includes excessive details about previous studies; it could be more concise. - Some sentences are overly theoretical without direct references (e.g., the hydraulic effect of the diaphragm). Each claim should be supported by an appropriate reference. - The following studies can be used in your manuscript, with suggestions on where they might fit: - Naderi, A., Shaabani, F., Esmaeili, A., Salman, Z., Borella, E., & Degens, H. (2019). **Effects of low and moderate acute resistance exercise on executive function in community-living older adults.** *Sport, Exercise, and Performance Psychology*, *8*(1), 106. Can be cited in the Introduction when discussing the cognitive benefits of short-term resistance exercise in older adults, and in the Discussion when interpreting findings related to exercise and executive function. - Naderi, A., Aminian‐Far, A., Gholami, F., Mousavi, S. H., Saghari, M., & Howatson, G. (2021). **Massage enhances recovery following exercise‐induced muscle damage in older adults.** *Scandinavian Journal of Medicine & Science in Sports*, *31*(3), 623-632. Suitable for the Introduction when emphasizing non-pharmacological recovery strategies, and in the Discussion when addressing interventions to reduce exercise-induced fatigue in older adults. - Naderi, A., Goli, S., Shephard, R. J., & Degens, H. (2021). **Six-month table tennis training improves body composition, bone health and physical performance in untrained older men; a randomized controlled trial.** *Science & Sports*, *36*(1), 72-e1. Can be included in the Introduction to highlight the benefits of long-term, multidimensional training programs on body composition, bone health, and performance, and in the Discussion when interpreting improvements in physical performance outcomes. - Naderi, A., Rezvani, M. H., Shaabani, F., & Bagheri, S. (2019). **Effect of kyphosis exercises on physical function, postural control and quality of life in elderly men with hyperkyphosis.** *Iranian Journal of Ageing*, *13*(4), 464-479. appropriate for the Introduction when introducing posture-specific exercise interventions for older adults, and in the Discussion when considering targeted interventions for kyphosis or postural control. | We sincerely thank the reviewer for this valuable and detailed feedback. In response, we have carefully revised the Introduction to improve clarity, scientific rigor, conciseness, and the overall structure. The revisions are summarized below:   - We have now explained the distinct mechanism and physiological contribution of DM in comparison with DB to clearly highlight how DM complements DB beyond respiratory effects (Introduction; pages 4–5, lines 83–93). - For all references involving younger adult populations, we have added an explicit statement of caution to acknowledge limited generalizability to older adults, while also using this to justify the existing knowledge gap and the need to investigate DB and DM specifically in community-dwelling older adults (Introduction; page 4, lines 79–82). - We have revised the hypothesis to be more specific, focused, and better structured, ensuring it reflects the study objectives without overextension or lack of prioritization (Introduction; page 5, lines 104–108). - To address the length and theoretical density of the section, we have condensed the discussion of previous studies and removed excessive background details to improve readability and conciseness (Introduction; pages 3–5). - We have ensured that all conceptual and physiological claims, including those related to intra-abdominal pressure and the diaphragm’s effect on postural systems (e.g., hydraulic effect via intra-abdominal pressure), are now directly supported with appropriate references (Introduction; page 4, lines 66–67). - We sincerely thank the reviewer for the suggested references. After careful review, we noted that most of the recommended studies examine interventions such as resistance training, table tennis, or massage, which address outcomes including cognitive performance, recovery, body composition, bone health, or general posture. While those topics are valuable, they do not directly correspond with the specific interventions or primary outcome of our study.   In line with the reviewer’s suggestion to acknowledge posture-targeted work, we incorporated the following study into the **Discussion section** to emphasize the importance of **targeted interventions for postural control** in older adults:  **Naderi, A., Rezvani, M. H., Shaabani, F., & Bagheri, S. (2019). *Effect of kyphosis exercises on physical function, postural control and quality of life in elderly men with hyperkyphosis*. Iranian Journal of Ageing, 13(4), 464–479.** (Discussion; Page 19, lines 410-412)  This citation enables us to recognize relevant posture-focused evidence while preserving the scope of our study, which specifically investigates DB and DB+DM as core techniques and their effects on balance, gait, fear of fall and quality of life related outcomes in community-dwelling older adults. |
| **Methods – Study Design**     - Line 153: The control group received no intervention, which may introduce attention bias. - Lines 32, 186: Follow-up was only 2 weeks, which is insufficient.      - Line 116: Trial registration number is not reported in the main text.   **Methods – Participants and Randomization**   - Lines 129–130: Sample size calculated with medium effect size, but actual power is low. - Line 127: Excluding participants with BMI >30 limits generalizability. - Line 135: Random sequence generation details are missing. - Line 133: The exact numbers of screened vs. eligible participants are not reported.   **Methods – Intervention**   - Lines 139, 145: Total intervention dose (20 minutes) is very low. - Lines 145–150: Actual difference between DB and DB+DM is only 3 minutes of DM. - Nowhere: Adherence rate is not reported.      - Nowhere: Number of missed sessions is not reported.      - Some intervention details (e.g., DM execution with figure-8 pattern) are overly technical and could be summarized in a figure or table.      - Follow-up duration of only 2 weeks limits assessment of long-term effects. - Nowhere: Inter-rater reliability for mini-BEST is not reported.   **Methods – Statistical Analysis**   - Line 194: Using LOCF is outdated and may introduce bias. - Lines 190–196, S3 Table: No adjustment for baseline imbalance in FSS. - Only in S2 Table: Effect sizes (η²) are not reported in the main text. | We sincerely thank the reviewer for these important and constructive feedback. We have revisited the manuscript to ensure transparency and clarity, and the corresponding revisions are summarized below:   - We have now explicitly acknowledged the possibility of attention bias arising from no-contact control and have clarified the procedural steps taken during the trial period to minimize and monitor this influence (Page 8, lines 155–159). - We agree that a 2-week follow-up period is relatively short and may not capture the full long-term effects of the interventions. This has already been reported as a limitation in the manuscript. We have rechecked the section and further strengthened its clarity to emphasize the need for longer follow-up assessments in future studies (Page 20, lines 438–440). - The trial registration number (**TCTR20240520003**) was previously included in the Study Design section. We have now carefully checked its placement in the main text and highlighted it (Page 6, lines 117–118). - The effect size used for the sample-size calculation (f = 0.30) was derived from our pilot data, which also indicated an estimated 30% attrition rate. We acknowledge that this resulted in a modest statistical power, and we have now clarified and highlighted this explanation in the manuscript. (page 7, lines 131-134). Additionally, we have highlighted in the Limitations section that future studies with larger samples are needed to confirm and extend these findings. (page 18, lines 398-400). - Dear reviewer, the participants with a BMI >30 were excluded to ensure participant safety and reduce potential confounding factors. Higher BMI can affect posture, spinal alignment, and balance, which could bias the measurement of outcomes. To carefully recruit participants with comparable characteristics and minimize such bias, we applied this exclusion criterion. - Random sequence generation was conducted using a computer-generated randomization list, and allocation was concealed with opaque envelopes by an independent researcher. This information is provided in the manuscript and now highlighted (see Methods, Sample Size and Randomization section: page 7, lines 137-139) - The numbers of screened and eligible participants now have been revised in the manuscript: 60 individuals were screened, of whom 54 met the inclusion criteria and were randomized into the three study groups. This information is also highlighted in the text, and we have revised the CONSORT diagram as well to clear representation (Fig. 1). (page 7, lines 135-136) - The intervention dose was intentionally kept low to ensure safety and feasibility for our population of community-dwelling older adults with balance impairments. DB and DM require sustained concentration and controlled respiratory effort, which can be fatiguing for older adults, particularly at the beginning of training. Based on ethical guidance and observations from our pilot study, a shorter session duration was selected to avoid overexertion, ensure good participant tolerance, and reduce the risk of dropout.   We agree that the overall intervention duration might be low, and we have mentioned in recommendation for future studies to explore longer and progressively increased training protocols. (Discussion; page 21, lines 455-458)   - Dear reviewer, although the DM component in the DB+DM group adds approximately 3 minutes per session, its purpose was not to increase total treatment time but to introduce a distinct manual therapy technique targeting diaphragm mobility, rib cage excursion, and fascial tension. The session duration was intentionally kept similar between both group 1 and 2 to control for treatment time to avoid confounding while isolating the added effect of manual mobilization. - Adherence was closely monitored, and participants who missed more than two sessions were required to complete makeup sessions in accordance with our protocol. All participants who completed the intervention met this requirement, resulting in a 100% adherence rate among completers. We have now added this information to the Methods section of the manuscript. (Methods; page 9-10, lines 198-201) - Dear reviewer, we have now clarified that participants who missed more than two sessions were required to complete makeup sessions, and those who were unable to meet this requirement were excluded from the study (two participants per group). All participants who completed the intervention fulfilled the session attendance requirement, resulting in 100% adherence among completers. Therefore, no completers had missed sessions beyond those made up. This information has now been explicitly stated in the Methods section for transparency. (page 9-10, lines 198-201) - Dear reviewer, the DM procedure has been highlighted in the Methods section, and we have now clarified the figure-8 motion in the **S3 Fig** caption at the end of the manuscript to improve clarity. (Methods section: page 7, lines 149-152 - Fig caption; page 27, lines 692-695) - Dear reviewer, we have acknowledged that this short 2-week follow-up may not capture the long-term effects of the interventions and could reflect the carry-over effects. We have mentioned it in the limitations and highlighted it in the text (Discussion; page 20, lines 438-440) - We have now mentioned the inter-rater reliability of the Mini-BESTest in the Methods section under the Outcome Measures. (Page 9, lines 182-183) - Dear reviewer, we have clarified in the Statistical Analysis section that LOCF was applied only for the six participants who missed the follow-up assessment and was used solely to maintain the ITT sample. (Methods; page 10, lines 204-205) - We acknowledge that baseline differences were present only in FSS scores across groups. To address this, we have mentioned in the results section that interpretations focus on changes over time and the group × time interaction using mixed ANOVA, which appropriately reflects differential responses between groups regardless of baseline values.   Additionally, we have mentioned this as a limitation of our study in the text. (Results; page 13, lines 270-273 – Discussion; page 20, lines 436-438)   - Dear reviewer, we have now added the effect sizes (η²) for all outcomes in the main text of the Results section, alongside the p-values, to clearly indicate the magnitude of the observed effects. (Results; pages 11-13; lines 224-226, 247-250, 252-253, 266-268, 284-286, 288-290) |
| **Results (lines 197–282)**   - Lines 251–252, S3 Table: Baseline imbalance in FSS undermines fatigue-related results. - Lines 204–277: Numerical change from baseline (Δ) is not reported in the text. - Lines 201–202: Claim of interaction without reporting η². - Only in S3 Table: Exact p-values for pairwise comparisons not reported in the text. - Nowhere: No mention of not reaching MDC in TUG/mini-BEST. - Some results (e.g., 5xSTS) claim significance but pairwise analysis shows no difference; recommend clarifying statistical vs. clinical significance. - Some p-values are borderline (e.g., p=0.05) and require cautious interpretation. - Reporting of clinical effects (MDC, MCID) is limited; it should be provided for each main outcome. | We thank the reviewer for the constructive comments to enhance the clarity and interpretability of the Results section. In response, we have thoroughly revised the manuscript, and all modifications are summarized point-by-point as follows:   - We thank the reviewer for the comment. We acknowledge the baseline imbalance in FSS scores and have clarified in the Results section that, for fatigue, interpretations focus on changes over time and the group × time interaction using mixed ANOVA. (Results; Page 13, lines 270-273) - Dear reviewer, the numerical changes from baseline (Δ) for all outcomes are provided in the tables (S3 Table) for each group at all time points. As our study includes 7 outcome measures assessed across 3 groups and 3 time points, including all Δ values in the main text would make it overly long. To maintain clarity and readability, we have focused on reporting statistical significance and effect sizes in the text, while detailed numerical values and changes are available in the tables. (S3 Table) - Dear reviewer, we have now added the effect sizes (η²) for all outcomes in the main text of the Results section, alongside the p values, to clearly indicate the magnitude of the observed effects. (pages 11-13; lines 224-226, 247-250, 252-253, 266-268, 284-286, 288-290) - Dear reviewer, we have now carefully reviewed the Results section and ensured that the exact p-values for all pairwise comparisons are clearly presented in the text. These have also been highlighted for ease of reference in the revised manuscript (Page 10–14, lines 214–303). - The MDC value for TUG has already been mentioned in the Discussion. Additionally, we have now highlighted the MDC value for TUG and, upon the suggestion, added the MDC for the mini-BEST (Page 16, lines 334–336, 351). - Dear reviewer, we have clarified in the Discussion that, although a significant group × time interaction was observed for 5xSTS, the absence of pairwise differences indicates that these changes were not clinically meaningful in terms of lower extremity strength improvements (Discussion; Page 17, lines 360–362). - Dear reviewer, we acknowledge that a few p-values (e.g., p = 0.05) are borderline and have ensured that these findings are interpreted cautiously in the Results and Discussion sections. - We have now included the MDC for the mini-BEST, our primary outcome measure, in the Discussion. Additionally, we have highlighted the MDC for outcomes that did not show clinically meaningful changes, such as TUG, to provide context on the clinical relevance of our findings (Page 16, lines 334–336, 351). |
| **Discussion (lines 284–412)**   - Line 292: Claims of “promising effects” despite no post-treatment balance improvement. - Lines 303–304: Interpretation of delayed effect in mini-BEST is not supported by evidence. - Nowhere: Baseline imbalance in FSS is not discussed. - Lines 331–339: Comparison with younger adult studies lacks caution. - Nowhere: Cost, training requirements, and feasibility in clinical practice are not discussed. - Lines 409–410: Suggestion to increase duration without defining minimum effective dose. - Sections are long and repetitive, particularly regarding DB and DB+DM effects on TUG and GV. - Explanation of null effects (e.g., 5xSTS and mini-BEST post-treatment) could be strengthened with physiological rationale or exercise intensity considerations. - Discussion could be more concise with practical recommendations for clinical application and future research.      - Discussion of MCID/MDC limitations could be expanded to help readers distinguish statistical vs. clinical significance. | We thank the reviewer for the valuable and constructive suggestions regarding the Discussion section. We have now revised this section to improve the clarity of interpretation, and enhance the clinical relevance of our findings. The key revisions are summarized below:   - We have revised the text to clarify that DB and DB+DM may improve certain aspects of physical performance and well-being when combined with additional or more targeted interventions, avoiding overstatement of effects. (Page 15, lines 319-320) - Dear reviewer, we did not intend to imply a delayed treatment effect. To ensure clarity, we have revised the text to simply report that mini-BEST scores were not statistically significant immediately post-treatment, while a statistically significant difference was observed at follow-up. However, this change did not meet the 4-point MDC threshold and therefore lacks clinical significance. We have updated the Discussion to reflect this more cautious interpretation.   We have also mentioned the possibility of learning effect on performance-based measures such as the mini-BESTest, as repeated exposure to the test could influence participant scores (Discussion; page 15-16, line 330-336, page 20, lines 440-442)   - We have reported the baseline between-group imbalance in FSS scores, and our repeated-measures intention-to-treat analysis accounted for these initial differences. The observed intervention effects remained consistent over time, ensuring that the reported improvements are attributable to the interventions rather than baseline variability. Upon the suggestion, we have now explicitly addressed this baseline imbalance in the Discussion section. Additionally, we have mentioned this in the limitation as well (Discussion; page 18, lines 386-389; page 20, lines 436-438) - We have acknowledged the differences between our older adult participants and younger adults in the Discussion, noting that older adults may require additional or more intensive strength-specific exercises to achieve comparable improvements in LES. This point has now been highlighted in the text (page 17, lines 370-375). - We have now added a brief discussion regarding the practical considerations of DB and DM interventions, including training requirements, cost, and feasibility in clinical practice (page 20, lines 427–431). - We have revised the text to clarify that, while longer or higher-intensity interventions may be beneficial, the minimum effective dose of DB and DB+DM in older adults remains to be determined and warrants further investigation. (page 21, lines 454-458) - We have carefully revised the paragraph discussing TUG and gait velocity to remove redundancy and improve clarity. Specifically, we condensed repeated statements regarding improvements from baseline to post-treatment and follow-up, combined overlapping descriptions of TUG and GV results into a single cohesive section. (Discussion; page 16, lines 344-356) - We have now expanded the Discussion to provide clearer physiological and methodological explanations for the null or delayed effects observed in both balance (mini-BEST) and lower extremity strength (5xSTS).   Specifically, in the mini-BEST paragraph, we have clarified that DB and DM primarily enhance diaphragmatic activation, trunk proprioception, and postural alignment rather than providing the multisystem sensory–motor challenge required to improve complex dynamic balance performance. (Discussion; page 15-16, line 330-334)  In the 5xSTS section, we have added a detailed rationale highlighting that DB and DM are low-intensity interventions that do not provide sufficient mechanical load to stimulate lower extremity strength gains. We mentioned that age-related physiological changes reduce responsiveness to low-load interventions, which may explain why older adults did not exhibit the improvements previously reported in younger populations. (Discussion; Page 17 , line 367-375)   - Dear reviewer, we revised the discussion for greater conciseness and ensured to include clear practical recommendations for clinicians (e.g., feasibility, minimal equipment, and integration into rehabilitation programs) as well as specific directions for future research (e.g., higher intensity, longer follow-up, and comparative interventions).   We believe these revisions address the concerns and significantly improve the clarity, relevance, and applicability of the Discussion. (Discussion; page 20, line 427-431)   - We have expanded the Discussion to more clearly differentiate statistical significance from clinical significance by explicitly addressing the MDC/MCID limitations of outcomes that did not show meaningful improvement (e.g., mini-BEST and TUG). This revision helps readers interpret the practical relevance of our findings. (Discussion; page 15-21, lines 311-458) |
| **Tables and Figures**   - S1 Table: Gender not reported by group. - S3 Table: Δ from baseline column missing.      - S1 Fig: Inconsistency in number analyzed (CONSORT). - S3 Fig caption: Insufficient explanation for “90/90/90 breathing.” | - Dear reviewer, we have carefully checked the S1 Table to clearly report gender distribution for each group, including both counts and percentages for females and males. - We sincerely thank the reviewer for this valuable suggestion. We have now added the Δ (delta) column, representing within-group mean change from baseline to post-treatment and follow-up, as requested. The changes are reported as mean ± SD, and the 95% confidence intervals (95% CI) are included in the corresponding column of the revised S3 Table. We have also updated the table caption in the supporting information at the end of the manuscript to reflect these additions for clarity. (Table S3, Supporting information; Page 28, line 704-707) - Thank you for pointing out the inconsistency in Figure 1. We have revised Figure 1 to clearly distinguish between participants who completed the study and those included in the analysis. Each group now shows “Completed the study (n = 16)” followed by “Analyzed (n = 18; intention-to-treat)” in the final row, to transparently reflect participant attrition and the analysis approach.   Furthermore, this clarification has been incorporated into the manuscript and is highlighted in the “manuscript with tracked changes” under the **Statistical Analysis section** (page 9, line 192) In addition, the caption for Figure 1 has been revised accordingly and is highlighted in the **Supporting Information section** (page 26, line 675).   - The S3 Figure caption has been revised to provide a clear explanation of “90/90/90 breathing,” specifying that participants lie supine with hips, knees, and shoulders flexed at 90° to facilitate optimal diaphragmatic expansion and trunk stability. (Supporting information; page 27, line 692-693) |
| **Limitations (lines 393–412)**   - Nowhere: Baseline imbalance in FSS not mentioned as a limitation. - Line 393: Low dose not identified as a primary limitation. - Nowhere: Adherence rate not reported. - Nowhere: Possible learning effect in mini-BEST not discussed. | - We have now added a statement acknowledging the baseline imbalance in fatigue (FSS) scores as a study limitation and clarified how this may have influenced post-intervention outcomes. This has been incorporated into the revised manuscript. (Discussion; page 20, line 436-438) - Dear reviewer, we have now explicitly identified the low-dose nature of the intervention (twice-weekly sessions over 8 weeks) as a primary limitation affecting both generalizability and potential treatment magnitude. This clarification has been added to the Limitations section. (Discussion; page 21, line 454-458) - We have now noted in the Limitations section that adherence rates, although high among completers, were not explicitly reported and that this information would have provided additional context regarding intervention feasibility. - We have now included the potential learning effect associated with repeated administration of the mini-BESTest as a limitation, acknowledging that familiarization with the test may have influenced performance outcomes. (Discussion; page 20, lines 442-445) |
| **Conclusion (lines 413–419)**   - Line 414: Overstated claims of statistical improvement in balance and strength. - Lines 415–416: No mention of the lack of clinical significance for balance and TUG. | - Dear reviewer, we have now clarified in the manuscript that, although statistically significant improvements were observed in balance (mini-BEST) and TUG, these changes may not meet established thresholds for clinical significance. The revised text now clearly distinguishes between statistical significance and clinical relevance to ensure accurate interpretation of these outcomes. (Conclusion; page 21, lines 460-466) |
| **References**   - Line 528: Reference 35 (Cozean questionnaire) is from a non-official source. - Lines 586, 593: References 58 and 59 formatted incorrectly. | - Dear reviewer, we have replaced the reference with an official source now and highlighted it in the manuscript. (Methods; page 6, line 129) - We have corrected the formatting of References 58 and 59 to align with the journal’s reference style guidelines. |
| **Supplementary Information**   - S2 Table: η² for group × time not reported in a separate column. - S3 Table: 95% CI for changes not reported. | - Dear reviewer, we have carefully checked that now the η² for the interaction term (group × time) is reported in a separate column in **S2 Table**, along with F and p-values. This column provides the effect size for the group × time interaction for each outcome measure. - Dear reviewer, we have now added the Δ column, representing the within-group mean change from baseline to post-treatment and follow-up, as requested. The changes are reported as mean ± SD, and the 95% confidence intervals (95% CI) in the corresponding columns of the revised S3 Table. We have also updated the table caption in Supporting Information at the end of the manuscript to reflect these additions for clarity. (S3 Table, Supporting information; page 28, line 704-707) |

Regarding the suggestion to publish the protocol separately on protocols.io, the intervention procedures are already comprehensively described in the Methods section and visually illustrated in Supporting Figures 2 and 3, offering sufficient clarity and reproducibility for readers. Therefore, we have opted not to publish the protocol separately on protocols.io for this study. We remain open to doing so in future research involving more complex or multi-phase intervention protocols.

Kind regards,
Anong Tantisuwat, PT, PhD, MPA

Associate Professor

Deputy Dean for Academic Affairs and Education, Information Technology, Public Relations, and Corporate Image

Mobile: +6663 5856363

E-mail: [anong.ta@chula.ac.th](mailto:anong.ta@chula.ac.th), [anongt@gmail.com](mailto:anongt@gmail.com)
